# A Numerical Study of Topography and Roughness of Sloped Surfaces Using Process Simulation Data for Laser Powder Bed Fusion

**DOI:** 10.3390/ma17235955

**Published:** 2024-12-05

**Authors:** Beytullah Aydogan, Kevin Chou

**Affiliations:** 1 Department of Industrial Engineering, J.B. Speed School of Engineering, University of Louisville, Louisville, KY 40292, USA; kevin.chou@louisville.edu; 2 Bayburt University, Bayburt 69000, Turkey

**Keywords:** additive manufacturing, laser powder bed fusion, topography, surface roughness, process simulation

## Abstract

The simulation of additive manufacturing has become a prominent research area in the past decade. Process physics simulations are employed to replicate laser powder bed fusion (L-PBF) manufacturing processes, aiming to predict potential issues through simulated data. This study focuses on calculating surface roughness by utilizing 3D surface topology extracted from simulated data, as surface roughness significantly influences part quality. Accurately predicting surface roughness using a simulation remains a persistent challenge. To address this challenge, the L-PBF technique with two different cases (pre- and post-contouring) was simulated using two-step process physics simulations. The discrete element method was utilized to simulate powder spreading, followed by the Flow-3D melting simulation. Ten layers were simulated at three different linear energy density (LED) combinations for both cases, with samples positioned at a 30-degree angle to accommodate upskin and downskin effects. Furthermore, a three-dimensional representation of the melted region for each layer was generated using the thermal gradient output from the simulated data. All generated 3D layers were stacked and merged to consolidate a 3D representation of the overall sample. The surfaces (upskin, downskin, and side skins) were extracted from this merged sample. Subsequently, these surfaces were analyzed, and surface roughness (Sa values) was calculated using MATLAB. The obtained values were then compared with experimental results. The downskin surface roughness results from the simulation were found to be within the range of the experimental results. This alignment is attributed to the fact that the physics simulation primarily focuses on melt pool depth and width. These promising findings indicate the potential for accurately predicting surface roughness through simulation.

## 1. Introduction

The laser powder bed is one of the major additive manufacturing techniques that has the capability of fabricating complex geometries [1]. Despite the feasibility of creating intricate designs, the process is not free from defects. L-PBF holds enormous promise, but it also faces several challenges, such as limited output, unstable and non-repeatable processes, and poor mechanical qualities of the components due to porosity or poorly finished surfaces [2]. Surface quality is particularly challenging, especially on inclined surfaces [3]. Since surface quality affects the sample’s lifecycle undesirably [4,5,6,7]. One of the major contributors to surface quality is build inclination. Compared to the upskin surface, the downskin surface demonstrates lower surface roughness [5,8,9,10]. Upskin formation can simply consist of step edges and attached powder particles [5,11]. Improving the roughness of upward-facing surfaces in L-PBF is particularly challenging, as they typically exhibit greater roughness compared to traditional side surfaces [12]. The step edge effect, resulting from the conversion of CAD geometry into layers, significantly contributes to the high surface roughness of inclined surfaces, including both upskin and downskin surfaces [9,13]. The downskin surface quality, on the other hand, is primarily determined by attached powder particles [5,8,14]. Moreover, powder attachments increase with the melt pool size, leading to greater surface roughness [5,15,16]. Surface roughness also impacts the post-processing cost of products by requiring more time and steps to achieve the desired surface quality [17]. However, in certain applications, such as biomedical applications, surface roughness can be desirable [18]. Therefore, characterizing surface roughness plays a significant role in both academia and industry. There are various methods to measure surface roughness. One method is contact-based, using a stylus profilometer to obtain a 2D line scan of the surface, which may result in an inaccurate estimation of the surface area [19]. Another method is noncontact scanning using white light interferometry (WLI), which provides Sa values that offer a better estimation of 3D surface roughness [20,21].

Numerical studies have been conducted to investigate the effects of process parameters on surface roughness [15,22,23,24,25,26,27]. However, these studies are limited due to the simplified single-layer, single-track scan strategy. The simulation results can assist in analyzing experimental data, as they provide the melt pool dimensions for each layer under similar processing conditions. In an experimental study conducted by Ren et al. [22], the pre-contouring scans resulted in smoother vertical and lateral surfaces on Cu-Cr-Zn alloy samples, compared to those made using the post-contouring strategy [22]. The high-fidelity numerical simulation by the ray-tracing method was also performed by Ren et al. [22] to study the laser absorption during post- and pre-contouring scans of vertical samples. The simulation results indicated that, during the pre-contouring scans, the presence of powder particles enhanced the laser absorption with a multireflection phenomenon. Higher laser absorption led to better melting of the scanning area and generated a continuous and smooth track. During the post-contouring scan, the liquid melt zone migrates towards the powder zone, resulting in more partially melted powder particles attached to the scan track [22]. Considering the results from this recent study, the possibility of developing a multitrack, multilayer simulation for the pre- and post-contouring strategy can be explored using physics-based simulations. These simulations can investigate surface formation by studying the melt pool dynamics. Furthermore, numerical methods are utilized to derive meaningful data for surface formation. COMSOL (COMSOL, Inc., Burlington, MA, USA) was used to investigate anomalies in the melt pool temperature distribution at the downskin location of the melt pool [24]. However, the methods used are simplified, with the fluid dynamics of the melt pool ignored [24]. ANSYS Fluent (ANSYS Inc., Austin, TX, USA) is another commercial software utilized to build a melt pool model in the overhang region that takes the melt pool’s fluid flow into account [14]. In the design of both aforementioned products, the effective medium technique was employed. However, it is important to note that this method disregards fundamental physics aspects, such as free surface movement, laser multireflection, and recoil pressure. Consequently, while it offers advantages in terms of processing time and resource efficiency, the accuracy of simulated findings is limited. Le and Chou [5] utilized Flow-3D and LIGGGHTS to mimic the LPBF system, including free surface movement, laser multireflection, and recoil pressure. Furthermore, Le and Chou suggested that the temperature gradient of the simulated data can provide insights into particle attachment on the melted part [5]. Thus, simulated data can be utilized to assess surface roughness. This study aims to calculate surface roughness by utilizing 3D surface topology extracted from the simulated data. To achieve this, the laser powder bed fusion technique was simulated using process physics simulation. Two different cases were simulated: one with pre-contouring and one with post-contouring. Ten layers were simulated at three different linear energy densities (LED) combinations for both cases—samples positioned at a 30-degree angle to accommodate upskin and downskin effects. Furthermore, a three-dimensional representation of the melted region for each layer was generated using thermal gradient output of simulated data. All generated 3D layers were stacked on each other and merged to consolidate a 3D representation of the overall sample. Surfaces (upskin, downskin, and side skins) were harvested from this merged single sample. Subsequently, surfaces were analyzed, and surface roughness (S_a_ values) was calculated using MATLAB. These values were then compared with experimental results.

## 2. Materials and Methods

The coupled discrete element method (DEM) with LIGGGHTS (an open-source code) and Flow-3D-based simulations for the pre-and post-contouring strategy and the strategy with different parameters for both contour scans were designed to mimic LPBF. Detailed methodology can be found in [5,9,28,29]. The simulation domain and size are similar to a previous study [5]. The build plate dimensions 900 µm × 1200 µm were used for the DEM simulation of the first layer. The obtained powder layer of a given thickness (30 µm) was fed as the input to Flow-3D for laser irradiation and melting. The melted and solidified part of the surface was extracted to simulate the second and subsequent layers using the same coupled DEM and Flow-3D framework.

Ti6Al4V was utilized in this study due to its well-established use in space and aerospace applications, as well as its extensive application in industry, particularly in LPBF processes. Additionally, raster scan parameters (laser power, P = 170 W; scan speed, V = 1250 mm/s) remained the same as our previous study, as they were the optimum parameters for the LPBF process in the EOS M270 setup with Ti6Al4V alloy. The designed contouring scan process parameters of pre-contouring simulations are given in Table 1. To understand the difference between surfaces inclined at different angles, the simulation was performed by giving a 30° inclination angle. The LED, particularly the scan speed, was selected by considering the computation time. The inner contour was scanned first, followed by the outer contour, and then, the raster scan for every layer.

The post-contouring scan process parameters for three different cases is presented in Table 2, for the simulation with different process parameters for both the inner and outer contour scans. Cases 1 and 3 used a very high LED for the inner contour scan and a mid or low LED for the outer contour scan. Case 2 used a medium LED for the inner contour and low LED for outer scans, respectively. The LED (particularly the scan speed) was decided by considering the computation time for the simulation. The objective was to study the surface formation by analyzing the melt pool geometry and dimensions at multiple layers.

**Figure 1:**
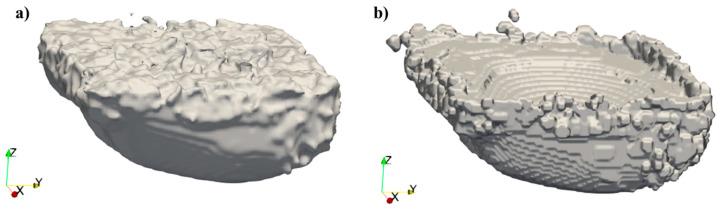
Three-dimensional representations of a single layer: (**a**) visualization of the melted region and (**b**) visualization of the temperature gradient. Both images were generated using the Flow3D post-processing environment.

The simulation results can be utilized to illustrate surface morphology. By employing the post-processing capabilities of Flow-3D, methods can be utilized to extract the molten region, thereby creating a surface representation and generating a comprehensive 3D model of the molten area for each layer. One approach involves obtaining a 3D representation of the melted region of the layer, which results in a smooth surface on the edges. Alternatively, utilizing the temperature gradient to generate a 3D representation has been explored. As suggested by Le and Chou [5], the temperature gradient may correlate with the powder attachment. Comparative images of both methods are presented in Figure 1. The differences between the methods are evident at the edges, which contribute to surface formation. These powder-like morphologies may indicate powder attachment, a major contributor to surface roughness, especially on the downskin. Consequently, the temperature gradient method is employed to obtain the 3D representation of a single layer.

**Figure 2:**
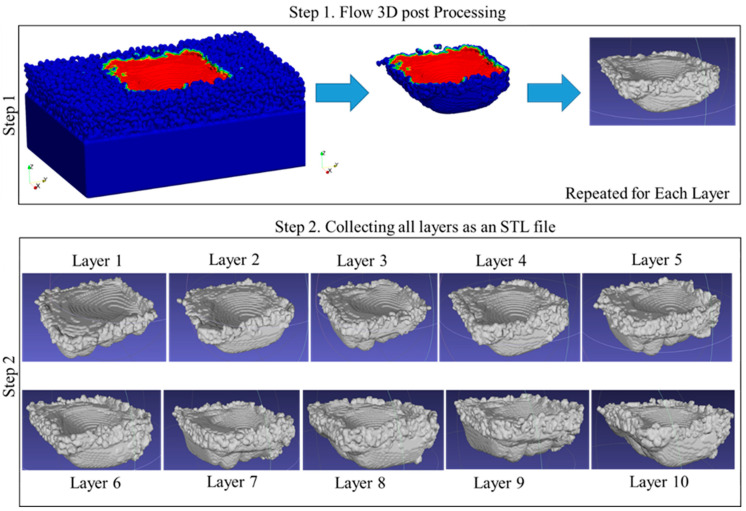
Gathering steps of the framework for the representation of the surface derived from the simulation results.

**Figure 3:**
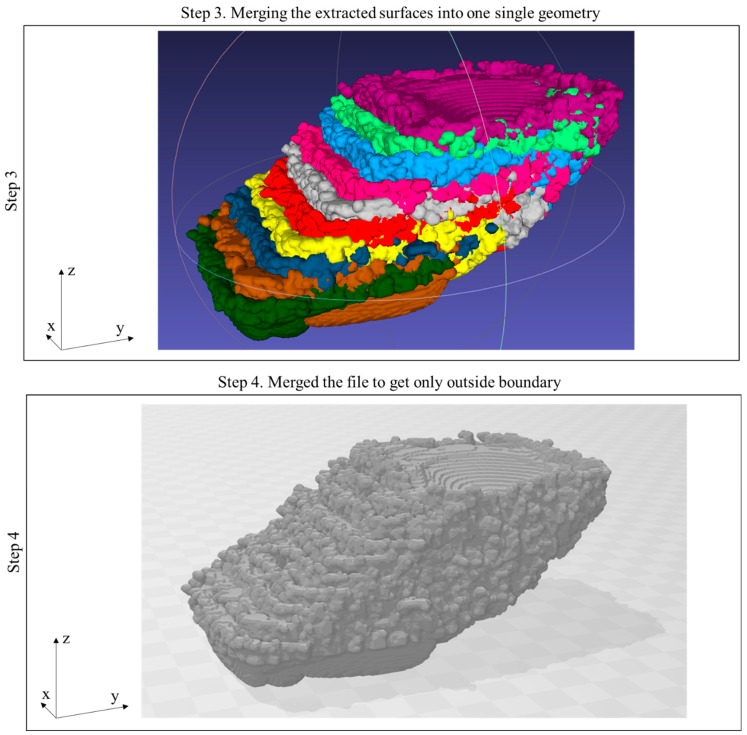
Merging steps of the framework for the representation of the surface derived from the simulation results.

**Figure 4:**
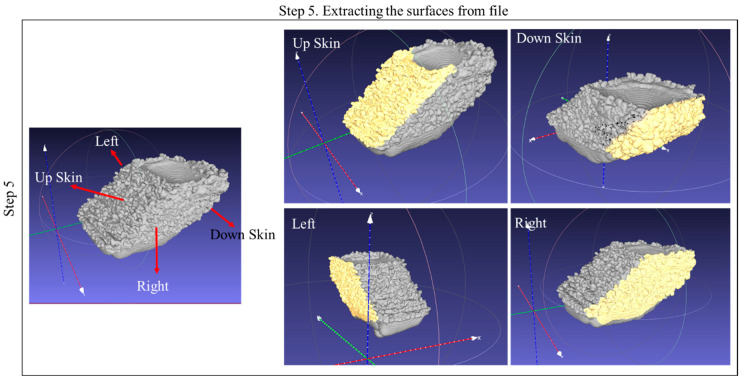
Extracting step of the framework for the representation of the surface derived from the simulation results.

The overall technique can be simplified into five steps. In the first step, the simulated layer is post-processed using Flow-3D Post software. Using the temperature gradient, a three-dimensional representation of the melted region is generated for each individual layer. Step 2 involves repeating this process for each layer and collecting the layers to form a 3D model. These steps are illustrated in Figure 2. In step 3, all layers are consolidated into a single file using MeshLab (2023.12) software, corresponding to the 3D model of the melted region. However, when merging is conducted, the merged sample contains all the interlayer connections. To isolate only the outer boundary of the merged file, Microsoft’s 3D Builder (20.0.4.0) software is utilized in step 4. These steps are illustrated in Figure 3. In the final step, the surfaces from the fully merged ten-layer simulation are harvested, including the upskin, downskin, and right and left side skins. This step is illustrated in Figure 4.

Harvested, individual files were loaded into MATLAB (2020a) for further analysis. After loading the STL files, scaling was applied to ensure that all reported sizes were consistent at the micron level. The files were manually cropped using numerical limits to isolate specific areas. In particular, the left- and right-side surfaces were rotated to perform additional crops and then rotated back to their original orientation. These cropping steps aimed to clean the surfaces related to the sides of the 3D object, thereby enabling more accurate calculations. Slight variations in cropping were applied to the samples to gather different values and improve accuracy. Three different crops were collected for each sample.

S_a_ values were calculated utilizing numerical approaches through Equation (1), enabling a comparison with experimental results. The calculation methods employed involve the initial displacement of the “z” plane to the mean position. Subsequently, the mean of the absolute “z” values yields the S_a_ values. To validate these findings experimentally, samples were fabricated using a powder bed fusion system, specifically an EOS M270. Ti6Al4V (Ti64) powder with a particle size range of 15 to 45 µm was utilized. The layer thickness was set to 30 µm, and a hatch spacing and beam diameter of 100 µm were employed. Samples were built with dimensions of 10 × 10 mm and a vertical height of 10 mm. Surface roughness was measured for three samples using white light interferometry (WLI) over an area of 2.7 × 1.3 mm. The experimental setup is explained in detail in Valiyakath Vadakkan Habeeb et al. [30]. Since the simulated areas resulted in considerably small surfaces, 30 random locations were selected that have an equivalent surface area to the simulated ones. This random selection of areas was coded in MATLAB (2020a) to ensure objective selection.

(1) $$S_{a}=\frac{1}{A}\iint \left|Z\left(x,y\right)\right|\;dxdy$$

## 3. Results

**Figure 5:**
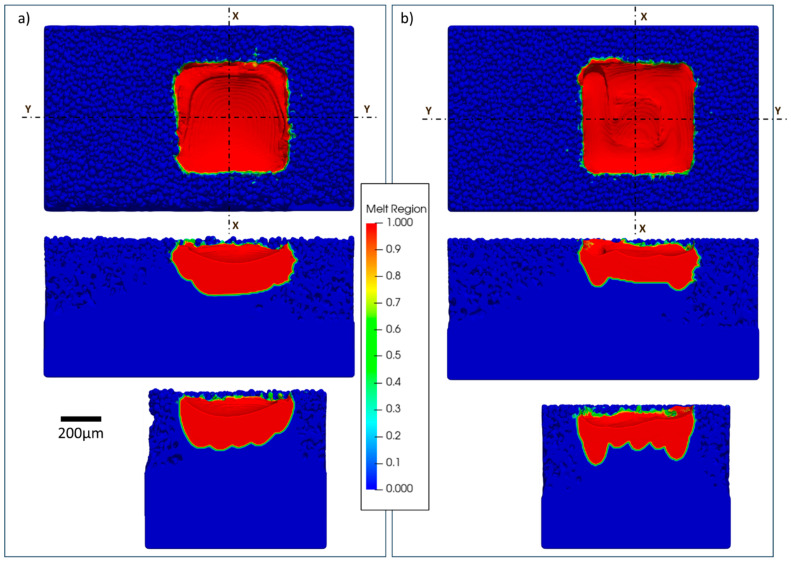
Melted region demonstration of the pre-contour high LED 10th layer (**a**) and post-contour high inner and low outer contour 10th layer (**b**).

Simulations were conducted up to the 10th layer for all cases. The representations of the melted regions for pre- and post-contouring simulations are illustrated in Figure 5. Notably, there are significant differences between the pre- and post-contouring strategies. The melted regions outside the boundary defining the sample’s outer walls were observed to be similar in both cases. However, the cross-sectional views of both cases demonstrated differences due to the order of the contouring strategy.

### 3.1. Simulation Outcome

**Figure 6:**
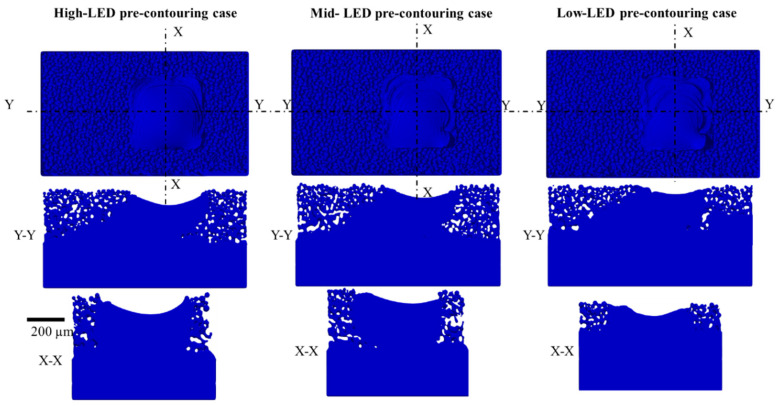
Pre-contouring strategy simulation results of the three cases at the 10th layer.

The pre-contouring strategy held up to the 10th layer for all three different LED cases. The comparison of results of the three simulation cases at the 10th layer is illustrated in Figure 6. A 30° angle is evident on the upskin region of the solidified domain. Additionally, the inclination angle at the downskin region is discernible in the Y-Y (sloping direction) cross-section. In the pre-contouring case, regardless of the energy density used, the shrinkage in the solidified region is observed at the center due to the subsequent raster scanning process.

**Figure 7:**
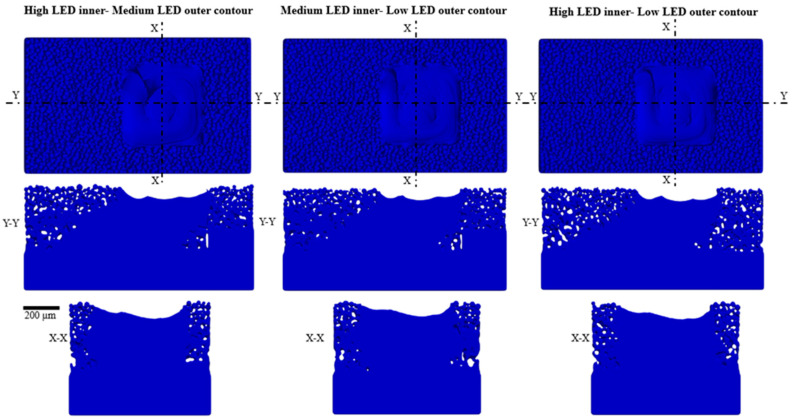
Post-contouring strategy simulation results of the three cases at the 10th layer.

However, the shrinkage of the melt pool changes in the post-contouring case. The results of the post-contouring simulation after the 10th layer are shown in Figure 7. Melt pool shrinkage is located on the contour side of the simulation for all post-contouring cases. The Y-Y cross-sectional image shows that the upskin surface clearly depicts the inclination angle, unlike the downskin surface, where the surface slope is not distinct.

### 3.2. Melt Pool Characteristic

**Figure 8:**
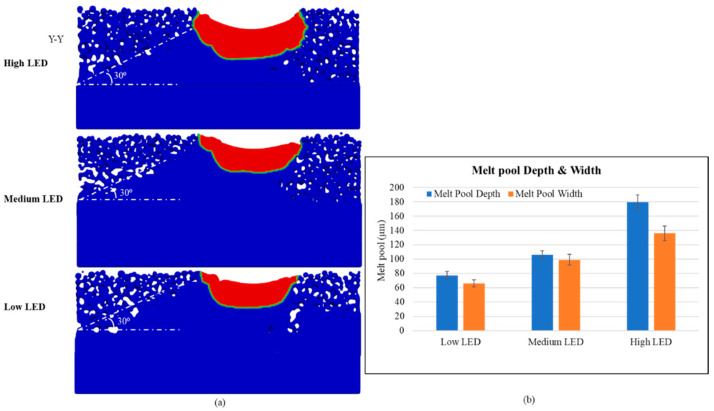
Simulated melt pool of the three cases of pre-contouring simulations at the Y-Y cross-section of the 10th layer (**a**) and measured melt pool dimensions (**b**).

The melt pool dimensions measured at the 10th layer are shown in Figure 8. As observed, the melt pool dimensions are smaller than those of the fifth layer. This phenomenon occurs, because the raster scan direction is parallel to the cross-section on the 10th layer compared to the 5th layer, resulting in fluctuations in the melt pool dimensions. The melt pool dimensions at the same location are consistent between the odd and even layers. The angle at the 10th layer is highly pronounced and visible.

**Figure 9:**
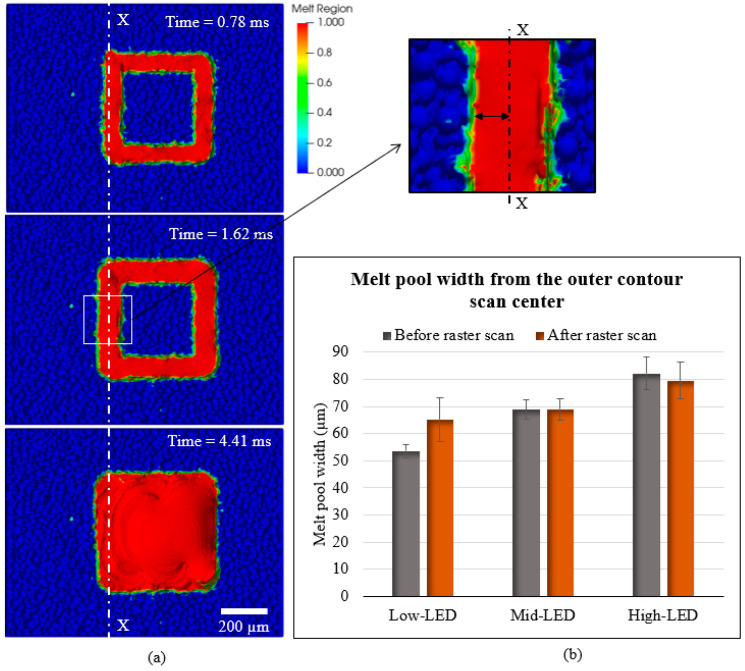
(**a**) Melt pool of low LED case simulation at different time steps. The first timestep (0.78 ms) is after the inner contour scan only, the second time step (1.62 ms) is after the inner and outer contour scans. The third timestep (4.41 ms) is after both pre-contour and raster scans. (**b**) Melt pool width from the center of outer contour scan before and after raster scan, from all three simulation cases.

The overlap of the raster scan beyond the pre-contour scan in the low LED case and better remelting of the contoured region in high and medium LED cases could be the reason for the aforementioned observation. To analyze the overlap of the raster scan onto the contour scan region, the melt pool width from the center of the outer contour scan on the upskin region before and after the raster scanning was measured at different time steps. Figure 9a shows the measurement on the low LED case, and Figure 9b illustrates the results from all three simulation cases.

For the medium LED and high LED cases, no significant disparities are observed before and after raster scanning. However, an increase in the melt pool width is observed in the low LED case after the raster scan. This outcome suggests that the raster scanning process remelts the contoured region. However, as the energy density and, hence, the melt pool width of the contour region are lower than the raster scan, it will override the contour region or visibly enlarge the effective melt pool. In other words, a substantial difference in LED levels between the raster and the contour scans, where the contour scan has a lower LED, results in the raster scan contributing to alterations in the surface topography. Hence, the results support the experimental observation.

**Figure 10:**
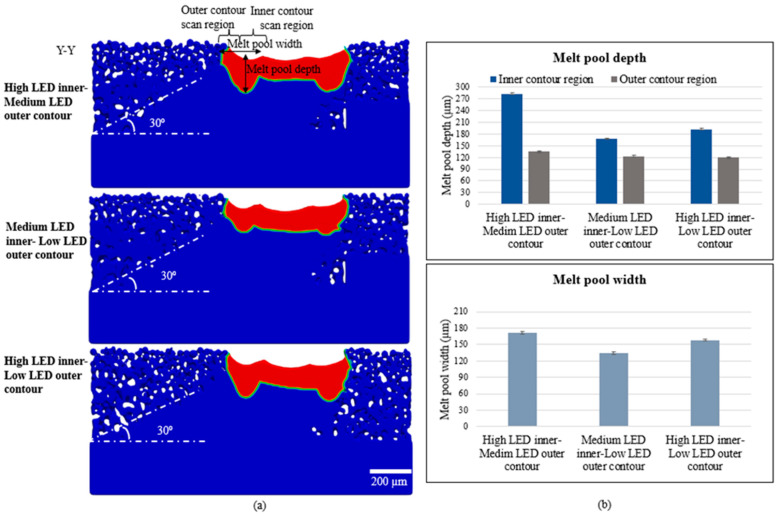
Simulated melt pool of three cases of post-contouring simulations at Y-Y cross-section (**a**) and measured melt pool dimensions (**b**).

The melt pool representation of the three simulation cases at the 10th layer is shown in Figure 10a. Melting along the upskin surface is better in the high LED inner contouring cases because of the larger melt pool and remelting using the outer contouring. However, the larger melt pool aids in more particle attachment on the downskin, hence not resulting in a sloped surface. The melt pool dimensions were measured after the 10th layer simulation and are shown in Figure 10b. The melt pool depth was measured at the inner and outer contour regions for all three cases. However, the width of the melt pool is not distinct due to continuous remelting and is not separately measured. The melt pool depth of the high LED inner-contoured cases is approximately same (~180 µm), irrespective of the outer contour LED used. The depth of the melt pool is slightly higher when the low LED outer contouring was used along with medium LED inner contouring, when compared to the case where it was coupled with high LED inner contouring. The nonmelted powder particles beside the melt pool that resulted from the medium LED inner contouring may have melted when the low LED outer contouring was used, which could be a reason for a distinct and deeper melt pool in this case. However, when high LED inner contouring is performed, the melt pool width is large, and the low LED outer contour scan may result in a melt pool contained within the larger inner contour region, which aids in remelting. That may be the reason for a smaller melt pool in the high LED inner/low LED outer contour case. The melt pool width data in Figure 10b support the analysis. The width of the melt pool created during the high LED inner/medium LED outer is approximately 15 µm larger than that of the high LED inner/low LED outer contour case, indicating a narrow or overlapped melt pool region during the low LED scan. The melt pool width is considerably low when medium LED inner contouring is performed. The melt pool dimension measurement on the downskin is not performed, as the melt pool shape and size vary at different layer simulations, due to the randomness of the powder particles underneath.

### 3.3. Thermal Gradient

**Figure 11:**
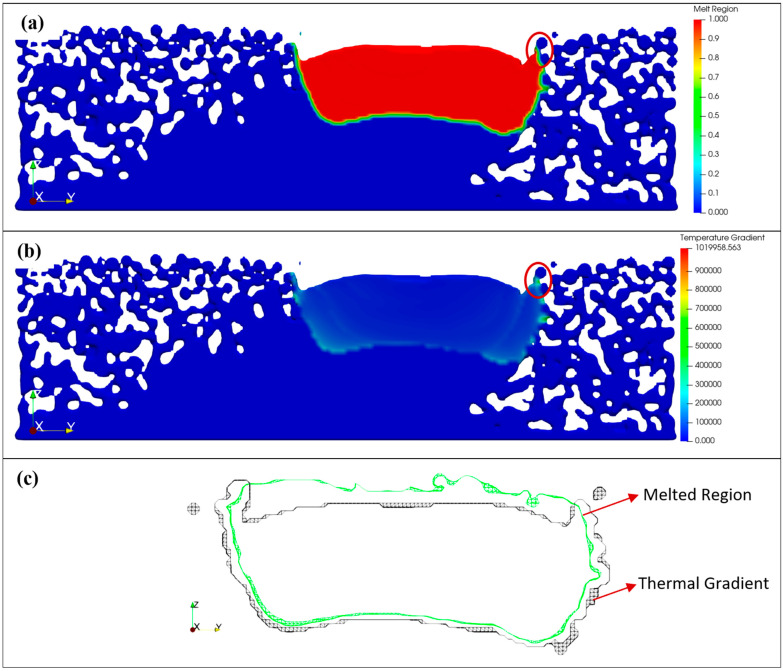
Comparison of thermal gradient and melted region results: (**a**) melted region representation, (**b**) thermal gradient representation within the fluid domain, and (**c**) comparison of melted region and thermal gradient, sliced from the 3D melted region and thermal gradient domain representation.

Simulation results were further investigated to define meaningful data for the surface formation. The melted region of the fluid domain was utilized to determine the melt pool characteristics of the simulations. Furthermore, the thermal gradient of the fluid domain was also analyzed. These results are demonstrated in Figure 11. The thermal gradient results and melted region results showed a similar trend in the fluid domain. Powder-like attachments are seen in both the melted region and thermal gradient, as indicated by the red circles. However, when the 3D representation of the melted region is compared, the difference is very dominant. Figure 11c shows the cross-sectional area of the melted region and the thermal gradient region. As seen in Figure 11c, the melted region representation is smaller and smoother than the thermal gradient method. Additionally, powder-like attachments disappear in the melted region representation. In contrast, the thermal gradient method demonstrates a more detailed contour on the surface formation. In particular, the sides that contribute to surface generation are more detailed, and powder-like attachments are mostly intact. Therefore, the thermal gradient method was utilized to generate the 3D representation of each layer.

### 3.4. Surface Topography and Roughness

**Figure 12:**
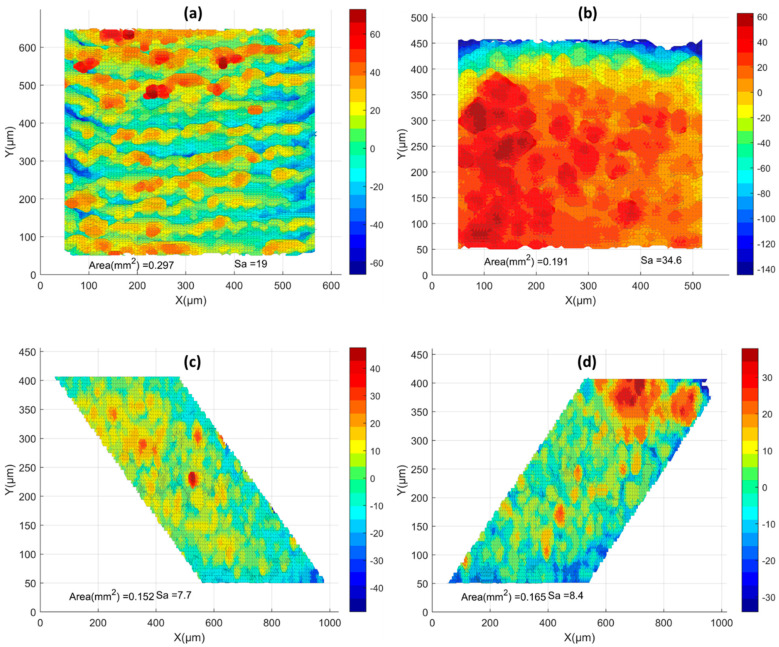
Pre-contour high LED surface morphology: (**a**) upskin, (**b**) downskin, and (**c**,**d**) side skins. MATLAB plots of the STL files were used for visualization.

**Figure 13:**
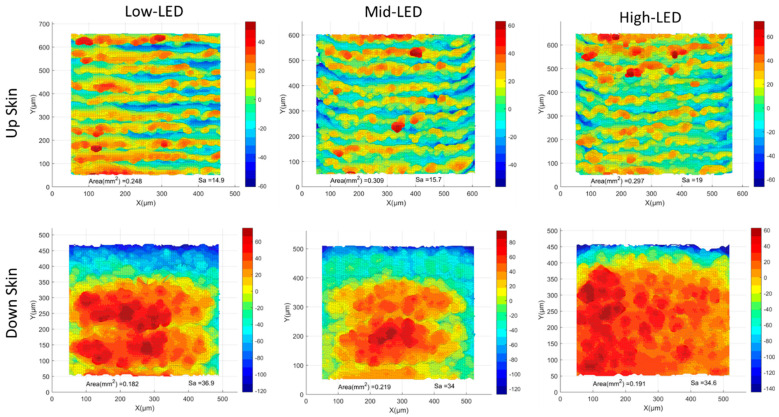
A comparison of surface topologies for all pre-contouring upskin and downskin conditions. MATLAB plots of the STL files were used for visualization.

**Figure 14:**
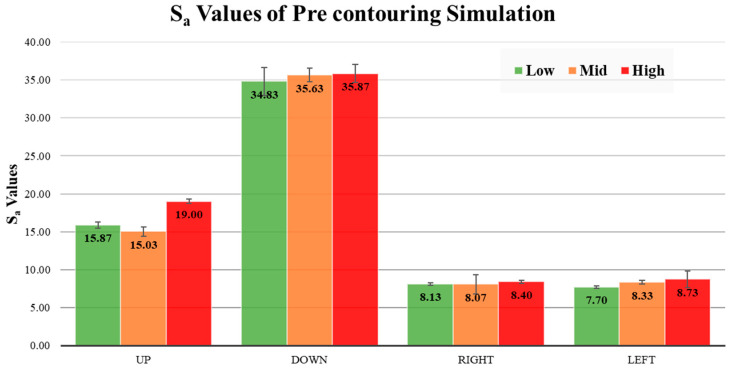
Pre-contouring simulation surface S_a_ values with different LED sets. Low refers to the low LED case, Mid to the medium LED case, and High to the high LED case on the contour region.

All surfaces were processed using the aforementioned methodology. Pre-contouring simulations were collected, and the surface topologies are presented in Figure 12. Additionally, a comparison of surface topologies for all pre-contouring upskin and downskin conditions is shown in Figure 13. Subsequently, the Sa values are illustrated in Figure 14. For the upskin results, the low LED showed lower S_a_ values compared to the others, while the high LED demonstrated higher roughness in the upskin. For downskin roughness, all values were higher compared to the upskin results, with the low LED and high LED showing similar results. The highest roughness was observed in the medium LED, with an S_a_ value of 19. Examining the side skins, both right and left demonstrated similar surface roughness, ranging from 7.7 to 8.73 for all LED cases. All side skins exhibited significantly lower surface roughness compared to the upskin and downskin. These surfaces consisted solely of the powder-like particles observed in the temperature gradient surface generation. In contrast, the upskin and downskin comprised step edges at a 30-degree angle. This makes the results for the upskin and downskin promising for the simulation of the surface quality. However, no visible trend was observed in either the upskin or downskin.

**Figure 15:**
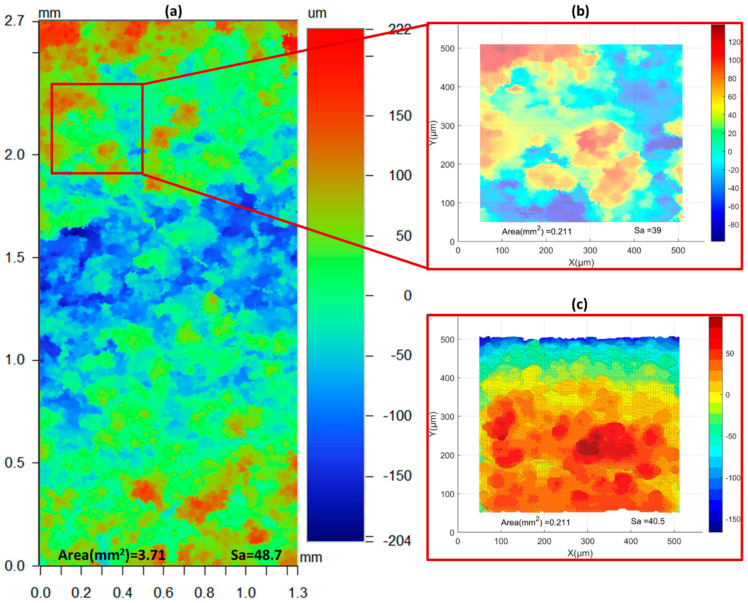
Post-contouring simulation with high inner and low outer LED parameters. Comparison of downskin surface size and roughness: (**a**) WLI image (A = 3.71 mm^2^, Sa = 48.7), (**b**) subsection of WLI image (A = 0.211 mm^2^, Sa = 39.0), and (**c**) simulated surface (A = 0.211 mm^2^, Sa = 40.5).

**Figure 16:**
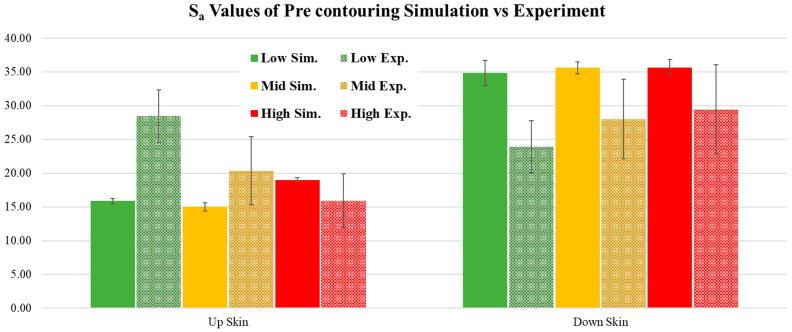
Comparison of roughness for the pre-contour case collected from simulation versus experiment. “Sim.” refers to the simulated surface results, and “Exp.” refers to the experimental results. Low refers to the low LED case, Mid to the medium LED case, and High to the high LED case on the contour region.

For verification purposes, the results were compared with their experimental counterparts. Since the experimental surface roughness images are significantly larger than the simulated ones, the WLI images were subset for a more accurate comparison. The differences are illustrated in Figure 15. Figure 16 illustrates the comparison between experimental and simulation surface roughness. For the upskin comparison, the simulation results indicate a reverse trend compared to the experiment. While the highest roughness in the experiment is observed in the low LED case, the simulation results showed low surface roughness for the low LED. The experimental results for the high LED showed the lowest surface roughness; whereas, the simulation counterpart showed the highest surface roughness compared to other simulated upskin results. The simulated surface results for the upskin were not close to the experimental ones. The main reason for this discrepancy may be the simulation physics. The upskin consists of the edge of the simulation surface and step-edge accumulation, while most simulations utilize the melt pool geometry to define the simulation characteristics. The upskin is more related to the bead height than to the melt pool depth and height. Conversely, the downskin region is highly correlated with the melt pool geometry. This results in the simulation showing promising results for surface roughness. All downskin results were within the standard deviation. All simulation results showed slightly lower S_a_ values compared to the experiments. In the low and medium LED cases, the results and trends were similar to the experimental results. However, the high LED case showed a slightly lower trend compared to the experimental trendline but still within the standard deviation range.

**Figure 17:**
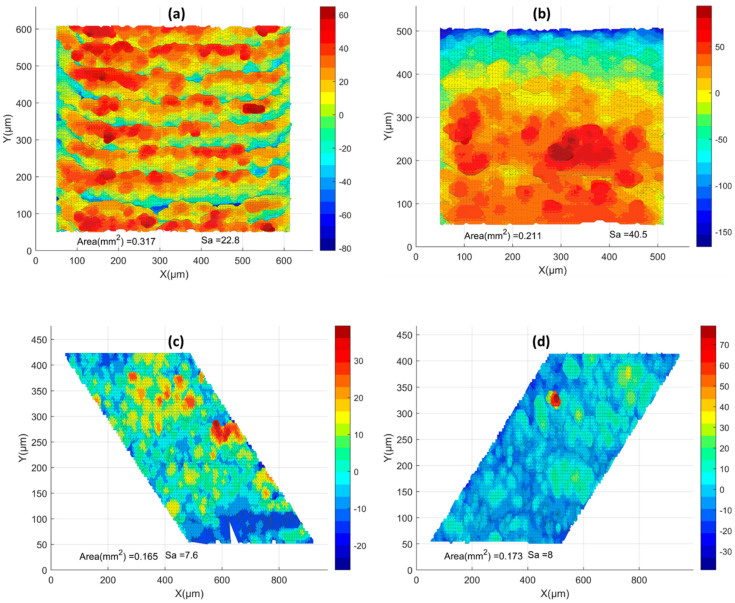
Post-contour high inner/low outer LED surface morphology: (**a**) upskin, (**b**) downskin, and (**c**,**d**) side skins. MATLAB plots of the STL files were used for visualization.

**Figure 18:**
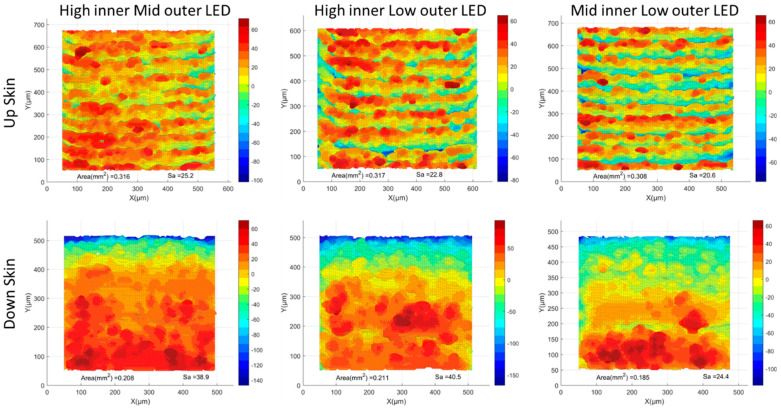
A comparison of surface topologies for all post-contouring upskin and downskin conditions. MATLAB plots of the STL files were used for visualization.

**Figure 19:**
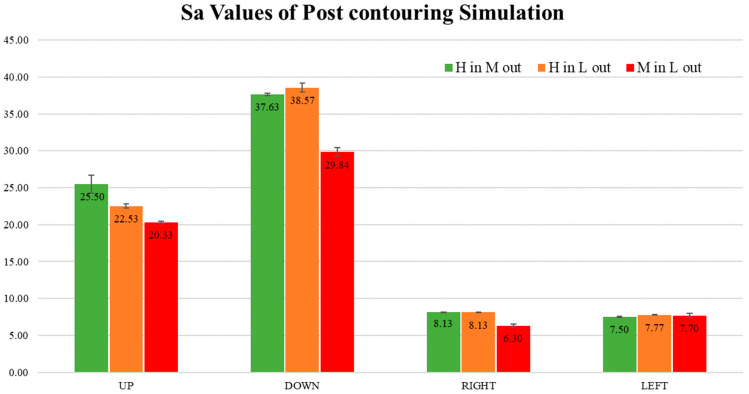
Post-contouring simulation surface S_a_ values with different LED sets. H in M out: high inner contour and medium outer contour. H in L out: high inner contour and low outer contour. M in L out: medium inner contour and low outer contour.

The post-contouring surface topology is presented in Figure 17. Additionally, Figure 18 shows a comparison of the surface topologies for all post-contouring upskin and downskin conditions. The simulation results are illustrated in Figure 19. In contrast to pre-contouring results, the post-contouring upskin results showed values almost similar to those of the downskin. The upskin S_a_ values fluctuated between 20.33 and 25.50, while, for the downskin, these values ranged from 29.84 to 38.57. There is no visible trend in the upskin values, but the downskin values demonstrated a clear trend. For the side skins, the results indicated very low surface roughness, with similar values observed between the left and right sides.

**Figure 20:**
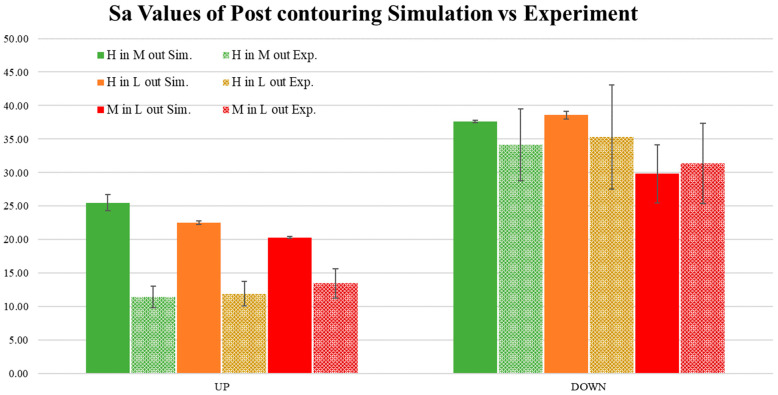
Comparison of roughness for the post contour case collected from simulation versus experiment. H in M out: high inner contour and medium outer contour. H in L out: high inner contour and low outer contour. M in L out: medium inner contour and low outer contour.

The results of post-contouring compared with the experimental values are depicted in Figure 20. The upskin experimental values were almost three times lower than the simulation results. The trend of experimental S_a_ values is not visible and appears reversed in the simulation of upskin values. Similar to the pre-contouring case, the upskin values do not match the experimental values. In contrast, the downskin values showed promising trends between the experiment and simulation. Similar to the pre-contouring cases, the simulation results for the downskin values were found to be within the standard deviation range of the experimental values.

## 4. Conclusions

The simulation data from Flow-3D were successfully utilized to depict 3D surface morphology. Two different cases with three different processing parameters were used in the simulation. In total, six different processing parameters were simulated, and their surfaces were extracted using the aforementioned method. The calculated S_a_ values were compared with the experimental values. The following conclusions were drawn from the study:

- In the pre-contouring case, the raster scan dominated the overall molten area, resulting in varying outcomes for the inclined area and melt pool dimensions across different layers. Also, regardless of the energy density used, the shrinkage in the solidified region was observed at the center due to the subsequent raster scanning process.
- In the post-contouring case, melt pool dimensions and sizes were found to be consistent throughout the simulation. The use of different LEDs showed promising molten geometry for the downskin formation.
- The high LED at the inner contour region significantly contributed to the formation of the upskin surface due to its deep and wide melt pool. As a result, the remelting of the contour region by the outer contour scan with a comparatively lower LED had minimal impact on the upskin surface formation, despite its role in remelting and smoothing the surface.
- The upskin surface roughness did not correspond to the experimental results across all cases and parameters. This discrepancy can be attributed to the upskin’s composition, which includes the edge of the simulation surface and step-edge accumulation. Most simulations utilized melt pool geometry to define their characteristics. However, the upskin was more closely related to the bead height than to the melt pool depth and height. Incorporating the bead height and geometry into the simulation may lead to improved results.
- The downskin S_a_ values showed a promising match with the experimental Sa values for all six parameters. This alignment may be attributed to the physics simulation’s focus on the melt pool height and width, which favorably influenced the downskin surface values.
- All side skins exhibited very similar results across all cases and parameters. Also, the side skins’ representation indicated that the temperature gradient method results in some level of powder-like attachments. Consequently, further studies may yield improved outcomes.
- Although the utilized physics simulation was limited by assuming powder as a solid–liquid, which excluded powder interaction, the thermal gradient approach resulted in reasonable surface roughness values.

It should be noted that the simulated sample represents a very small section of the real sample, which may affect the results due to the limited area. The downskin simulation roughness results are promising for future studies. This study could be expanded to include a larger simulated area for more comprehensive analysis. Additionally, different materials may yield varying results in both simulations and experiments, and further research could explore the effects of different materials on these outcomes.

## Figures and Tables

**Table 1:** Process parameters for simulation with the pre-contouring strategy for the inner and outer contour scans.

Simulation Case	Laser Power, P (W)	Scan Speed, V (mm/s)
High LED	200	1250
Medium LED	150	2000
Low LED	100	2000

**Table 2:** Process parameters for simulation with two different parameters for the inner and outer contour scans.

Case No	Inner Contour		Outer Contour	
	Power, P (W)	Scan Speed, V (mm/s)	Power, P (W)	Scan Speed, V (mm/s)
1	200	1250	150	1250
2	150	1250	100	1250
3	200	1250	100	1250
	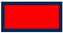 High LED 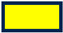 Medium LED 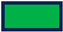 Low LED			

## Data Availability

The original contributions presented in the study are included in the article, further inquiries can be directed to the corresponding author.
